# The impact of bilateral anodal transcranial direct current stimulation of the premotor and cerebellar cortices on physiological and performance parameters of gymnastic athletes: a randomized, cross-over, sham-controlled study

**DOI:** 10.1038/s41598-023-37843-1

**Published:** 2023-06-30

**Authors:** Sajjad Anoushiravani, Jaber Alizadehgoradel, Asgar Iranpour, Omid Yousefi Bilehsavar, Asghar Pouresmali, Michael A. Nitsche, Mohammad Ali Salehinejad, Mohsen Mosayebi-Samani, Maryam Zoghi

**Affiliations:** 1 Department of Sports Physiology, Faculty of Educational Sciences and Psychology, University of Mohaghegh Ardabili, Ardabil, Iran; 2 Department of Psychology, Faculty of Humanities, University of Zanjan, Zanjan, Iran; 3 Department of Family Health, Social Determinants of Health Research Center, Ardabil University of Medical Sciences, Ardabil, Iran; 4 Department of Psychology and Neurosciences, Leibniz Research Centre for Working Environment and Human Factors, Dortmund, Germany; 5 University Hospital OWL, Protestant Hospital of Bethel Foundation, University Clinic of Psychiatry and Psychotherapy and University Clinic of Child and Adolescent Psychiatry and Psychotherapy, Bielefeld University, Bielefeld, Germany; 6 Discipline of Physiotherapy, Institute of Health and Wellbeing, Federation University, Victoria, Australia

**Keywords:** Psychology, Neurophysiology

## Abstract

Professional sports performance relies critically on the interaction between the brain and muscles during movement. Transcranial direct current stimulation (tDCS) is a noninvasive brain stimulation technique which modulates cortical excitability and can be used to improve motor performance in athletes. The present study aimed to investigate the effect of bilateral anodal tDCS (2 mA, 20 min) over the premotor cortex or cerebellum on motor and physiological functions and peak performance of professional gymnastics athletes. Seventeen professional gymnastics athletes participated in a randomized, sham-controlled, crossover study. In this study, we assessed the efficacy of two anodal tDCS protocols (2 mA, 20 min) with stimulation over the bilateral premotor cortex or cerebellum with the return electrodes placed over the opposite supraorbital areas. Power speed, strength coordination, endurance, static and dynamic strength, static and dynamic flexibility, and rating of perceived exertion were measured before and immediately after tDCS interventions (bilateral anodal tDCS over premotor cortices, anodal tDCS over the cerebellum, and sham tDCS). Additionally, physiological muscle performance parameters, including maximum voluntary isometric contraction (MVIC) of upper body muscles, were assessed during tDCS. Bilateral anodal tDCS over the premotor cortex, compared to anodal tDCS over the cerebellum and sham tDCS conditions, significantly improved power speed, strength coordination, and static and dynamic strength variables of professional gymnastics athletes. Furthermore, bilateral anodal tDCS over the cerebellum, compared to sham tDCS, significantly improved strength coordination. Moreover, bilateral premotor anodal tDCS significantly increased MVIC of all upper body muscles during stimulation, while anodal tDCS over the cerebellum increased MVIC in only some muscles. Bilateral anodal tDCS over the premotor cortex, and to a minor degree over the cerebellum, might be suited to improve some aspects of motor and physiological functions and peak performance levels of professional gymnastics athletes.

Clinical Trial Registration ID: IRCT20180724040579N2.

## Introduction

Professional sports performance relies heavily on the interaction between the brain and muscles during movement^1^. Sports performance involves various physical, physiological and psychological factors that determine speed, coordination and fatigue^2^. Besides unspecific health- and fitness-related aspects, such as strength, speed, agility, reaction time, balance and coordination, also the athlete's technique and competence level in specific motor skills play an important role^3^. Improving exercise performance is aimed at both, individuals seeking health and fitness (i.e., non-athletes), and those who wish to improve athletic performance (i.e., athletes)^4^. Learning motor skills plays an important role in improving sports performance, which is generally defined as a set of processes aimed at learning and refining new skills by practice^5^, and associated with functional and structural changes in a distributed brain network for example the primary motor area^6^. Frontoparietal networks are critical for motor learning, and performance, and play key roles in motor skill consolidation, and storage^7^. The motor system is plastic, allowing rapid acquisition of new movements and adaptation of existing movement sequences to meet environmental demands^8^. Accurate execution of a goal-directed action requires the integration of visual and somatosensory information into appropriate motor commands^9^. With this information transmitted by multiple sensory systems, the frontal brain networks plan and produce the appropriate motor commands, and the premotor cortex forms an important node in this neural network^10^. The important role of the PMC as a critical node in the neural network that implements the control and learning of goal-directed actions has been demonstrated in healthy individuals and individuals with brain damage^9^. It is also known that the cerebellum is involved in motor learning and temporal processing of movements^11^. Previous research has shown that motor training can modulate cerebellar activation. For example, Koeneke et al. found that skilled keyboard players showed less cerebellar activity than control participants during unimanual and bimanual finger movements, which hints at less effort required in case of higher skill levels^12^.

Cortical plasticity plays a key role in motor learning, and leads to the improvement of motor skills. Non-invasive brain stimulation (NIBS) techniques^13^ are suited to induce neuroplasticity in the human brain, and have been used to modulate the plasticity of the human motor cortex to facilitate motor learning^14,15^. These effects may reflect underlying synaptic mechanisms involving long-term potentiation (LTP) or long-term depression (LTD)^13^ One of these plasticity-inducing NIBS techniques is transcranial direct current stimulation (tDCS)^16^. tDCS involves applying a weak electrical current to the brain via the scalp by conductive electrodes. This electrical current alters the pattern of neuronal excitability dependent on current flow direction. At the macroscale level, stimulation with the anode positioned over the target area enhances cortical excitability, while cathodal stimulation has antagonistic effects with standard protocols^17,18^. TDCS induces glutamatergic plasticity, while GABA reduction also generated by tDCS might gate these effects^19^. Because tDCS modulates not induces cerebral activity, its effects also require spontaneous cortical activity^20^. Due to its potential to noninvasively induce acute, and outlasting cortical excitability alterations, tDCS raised interest in the field of motor performance improvement^15^. The concept of application of tDCS for improving motor performance in athletes comes from the importance of plasticity for motor learning^21^ and the fact that tDCS improves motor learning in healthy individuals^22–24^, and patients^25–27^. Moreover, it has been shown that—when applied in association with motor performance not related to learning—anodal tDCS enhances primarily cortical networks activated by motor activity^28^, and might thus improve also motor performance not related to learning.

In accordance, Lattari et al. showed that bilateral tDCS over M1 with 2 mA for 20 min significantly increases power-related task performance such as vertical jump ability^29^. Similar to this finding, Codella et al. showed that bilateral tDCS over M1 increases the strength of lower limb muscles^30^. Grosprêtre et al. report that anodal tDCS over M1 increased supraspinal and spinal excitability and improved jump performance in intermediate level parkour athletes^31^. Importantly in connection with the present study, Kaminski et al. (2016) provided evidence for the ability of anodal tDCS over the M1 leg area to improve dynamic balance performance in the lower limbs^32^. Beyond the M1 area, stimulation of the dorsal premotor cortex (PMd) using transcranial magnetic stimulation (TMS) increased reaction time in another study, and thus supports its critical role in motor planning^33^. In this connection, PMd neurons serve two main roles: (a) integration of sensory information into motor commands and (b) specification of movement parameters such as movement amplitude, direction, and speed^10^. Moreover, premotor anodal tDCS has been shown to improve motor performance after stroke^34^. While most tDCS applications in sports have focused on M1and premotor areas^35,36^ so far, the cerebellum is increasingly considered as an additional target^37,38^. Transcranial direct current stimulation of the cerebellum (ctDCS) has been explored as an alternative stimulation site for tDCS to promote motor learning^39^. Previous studies have shown that anodal tDCS over the M1 improves motor learning in a number of fine motor skill tasks in healthy subjects^40,41^. On the other hand, targeting the cerebellum is important because it controls and coordinates complex movements and is important for adapting movements to changes in feedback. The cerebellum receives sensory and motor information from descending cortical pathways and ascending peripheral pathways. It also has connections to the parietal, premotor, and frontal cortex^42^.

Preliminary evidence suggests the efficacy of bilateral anodal stimulation over both motor cortices to improve endurance performance in healthy subjects^43^. In this study, however, bilateral was not compared to unilateral stimulation. Although tDCS over the premotor cortex suggest an effectiveness of this protocol for improving motor skills, no studies have applied bilateral stimulation over this area to improve motor skills of athletes. Moreover, no respective studies have yet been conducted in gymnastics. Gymnasts have to achieve high levels of strength, flexibility, and coordination in order to effectively perform a wide variety of complex acrobatic skills^44,45^. The beauty of any gymnastic dramatic move is based on the seemingly effortless coordination of the muscles involved in movements^46^. Using NIBS in gymnastics athletes might improve movement performance via excitability, and plasticity enhancement. Specifically, cerebellar and premotor regions are crucial for gymnastics because of their role in motor coordination^47^. The present study aimed to provide new insights in the effects of tDCS in gymnastics athletes. Accordingly, in this registered, randomized, cross-over, sham-controlled trial we aimed to (1) investigate the effect of bilateral anodal tDCS (2 mA, 20 min) over the premotor cortex on physiological and performance parameters of professional gymnastics athletes, (2) investigate the effect of bilateral anodal cerebellar tDCS (2 mA, 20 min) on physiological and performance parameters of these athletes, (3) compare the effectiveness of stimulation of these two areas (cerebellar and premotor) on sports performance of gymnasts.

We hypothesized that bilateral anodal tDCS over the premotor cortex and cerebellum significantly improves the physiological and performance parameters of professional gymnastics athletes via its impact on strength, and coordination.

## Materials and methods

### Participants

Twenty professional gymnasts (mean age = 21.05 ± 2.04) were recruited from Ardabil city in Iran. Three participants did not complete all experimental conditions because of sports training programs for competitions, and the final analysis was conducted on 17 athletes. The required sample size analysis was calculated by G* Power software for an effect size based on previous tDCS studies conducted to alter sports performance^48^ (*f* = 0.4), an alpha error of *α* = 0.05, a *power of* 0.80, and an ANOVA as primary statistical test, and resulted in an N = 18. We added 2 participants to compensate for 10% dropouts rate. The demographic information of the participants is shown in Table 1. The inclusion criteria were: (1) professional level of performance by participating in the national championship, (2) no current or past history of epilepsy, seizures, or head injury, (3) and being 18–25 years old. The study was performed in accordance with the latest version of the Declaration of Helsinki ethical standards, confirmed by the Ethical Committee of the Ardabil Medical Sciences University and then registered in the International Clinical Trials Registry Platform with identifier IRCT20180724040579N2. All participants signed a written informed consent form before participation. Also, the experimental protocol was approved by a scientific committee of the University of Mohaghegh Ardabili.

**Table 1:** Demographic data.

Variable	Value
Sample size (*n*)	17
Age—mean (SD)	21.05 (2.04)
Sex—male (female)	17 (0)
Marital status—single (married)	15 (2)
Education—diploma (BA)	14 (3)
Height—mean (SD)	175.41 (2.85)
Weight—mean (SD)	71.23 (2.27)
BMI—mean (SD)	23.16 (0.43)
Body fat percentage—mean (SD)	5.00 (0.88)
Fat mass—mean (SD)	3.57 (0.66)
Lean body mass—mean (SD)	67.60 (2.09)
Number of years in practice—mean (SD)	13.23 (2.43)
Hours practice per week—mean (SD)	10.35 (1.58)
Resting heart rate—mean (SD)	57.88 (3.14)
Maximal heart rate (HRmax value)—mean (SD)	198.94 (2.04)

### Demographic parameter assessment

All demographic and baseline physiological data such as age, sex, marital status, education, height, weight, body mass index, body fat percentage, fat mass, lean body mass, years of practice, hours of practice per week, and resting heart rate and maximal heart rate were recorded in the first session (Table 1). Subjects were asked not to eat or drink within 3 h before the anthropometric measures. A Seca Digital Scale (hyper model, China) and a wall-mounted height scale were used for body weight and height measurements, respectively. The average of three sequential measurements was used for anthropometric measurements. Body mass index was determined as the body mass divided by the square of the body height, and is expressed in units of kg/m^2^, resulting from weight in kilograms and height in metres. Heart rate measures were conducted using a Polar HR monitor device (Polar Electro, Kempele, Finland). To estimate the maximum age-related heart rate, age was subtracted from 220.

### Measures

#### Performance measures

**Figure 1:**
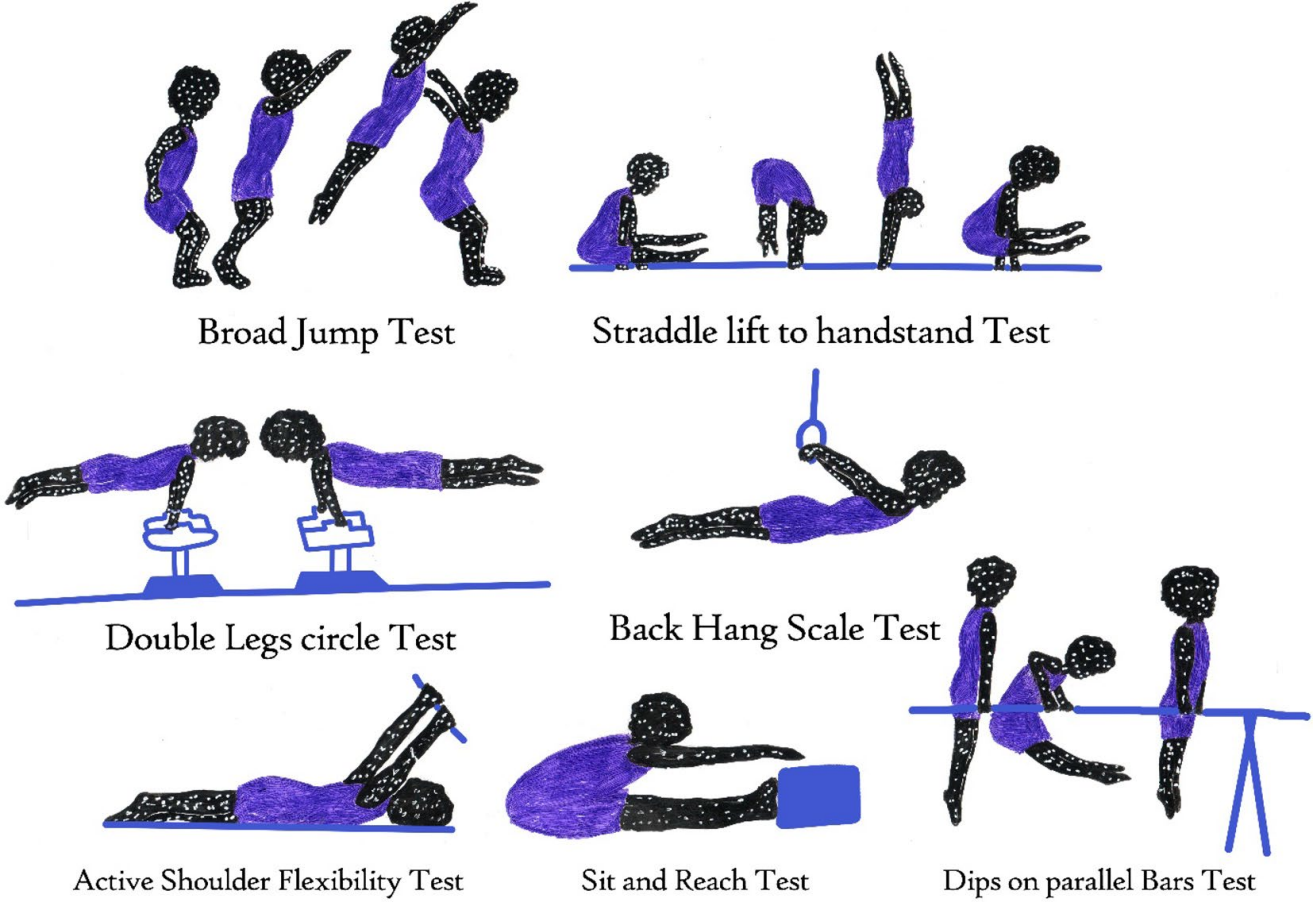
Gymnastics performance profile assessment. The measurement units of the Broad Jump Test (BJT), Active Shoulder Flexibility Test (ASFT) and Sit and Reach Test (SRT) were the centimeter value of the ruler from the zero point; The measurement units of the Straddle lift to handstand Test (SLHT), Double Legs Circle Test (DLCT) and Dips on Parallel Bars Test (DPBT) were the number of repetitions from the zero point. The measurement unit of the Back Hang Scale Test (BHST) was endurance in seconds.

In this study, the Broad Jump Test (BJT), Straddle lift to handstand Test (SLHT), Double Legs circle Test (DLCT), Back Hang Scale Test (BHST), Active Shoulder Flexibility Test (ASFT), Sit and Reach Test (SRT), and Dips on Parallel Bars Test (DPBT) were used to measure power speed and velocity coordination, strength coordination target and endurance, coordination endurance, static strength target and isometric coordination, dynamic flexibility target, static flexibility, and dynamic strength target and endurance, respectively (Fig. 1). Before performing the tests, each subject performed optional warm-up exercises for 5 min with an intensity of nearly 100 heart beats per minute.

##### Broad jump test (BJT)

The BJT is a test developed based on power speed target and velocity coordination. For performing the BJT, the subject stands at a line that is marked on the ground with the feet slightly apart. The subject takes off and provides a forward drive, using both feet with swinging the arms and bending the knees. The measurement unit of this test is centimeters (cm).

##### Straddle lift to handstand test (SLHT)

The SLHT is an advanced arm-balancing test that is performed based on strength coordination target and endurance. To perform a straddle press handstand, the first step is holding the handstand vertically, with legs extended out to either side of the body at a 45-degree angle condition, then the lower part of the body is pulled up with the power of the arms while aligning the pair of legs with the ground. The measurement unit of this test is the number of repetitions (Reps).

##### Double legs circle test (DLCT)

The DLCT is a measure of coordination endurance of gymnastic performance. Subjects keep the legs together at all times. To perform this test, subjects start off by leaning on both arms while the legs are leaning out and away from the body. As the subject swings hips and legs in a circle, one arm has to be lifted, and the body weight put to the other arm as the full leaning out legs are passed around the body in a range from 45 to 70 degrees. The measurement unit of this test is the number of repetitions (Reps).

##### Back hang scale test (BHST)

The BHST is a test based on static strength target and isometric coordination conditions. Participants perform first a static hold on the rings, and then they lower themselves from an inverted hang or inverted picked hang until the body is in an horizontal position with the face positioned towards the floor. The body should be fully straight extended, arms should be tight, wrists should be facing the floor, and the head must be in the middle position. The measurement unit of this test is stability of this position in seconds (s).

##### Active shoulder flexibility test (ASFT)

The ASFT measures dynamic flexibility target. To perform this test, the subject is lying on his stomach and holds a wooden bar at both ends with his hands, then raise the wooden bar directly above the head as much as possible. The examiner uses a tape to measure and record the distance between the center of the wooden bar and the ground. The measurement unit of this test is centimeters (cm).

##### Sit and reach test (SRT)

The SRT is a static flexibility test that specifically measures the flexibility of the lower back and hamstring muscles. The SRT test starts with sitting on the floor, then subjects stretch out their legs straight ahead. Subjects place the soles of the feet flat against a box, lock both knees, press the legs flat to the floor and put the hands on top of each other to reach forwards. After reaching, the subject holds that position for at least one-two seconds while the distance from the zero point of the flexibility measuring ruler installed on the board is recorded. The measurement unit of this test is centimeters (cm).

##### Dips on parallel bars test (DPBT)

The DPBT is based on a dynamic strength target and endurance condition. Subjects grab the parallel bars, jump up and straighten the arms, bending the arms while leaning forwards to the lowered body, dip down until the shoulders are below the elbows, lift the body up by straightening the arms and lock elbows at the top. The measurement unit of this test is the number of repetitions (Reps).

### Transcranial direct current stimulation (TDCS)

**Figure 2:**
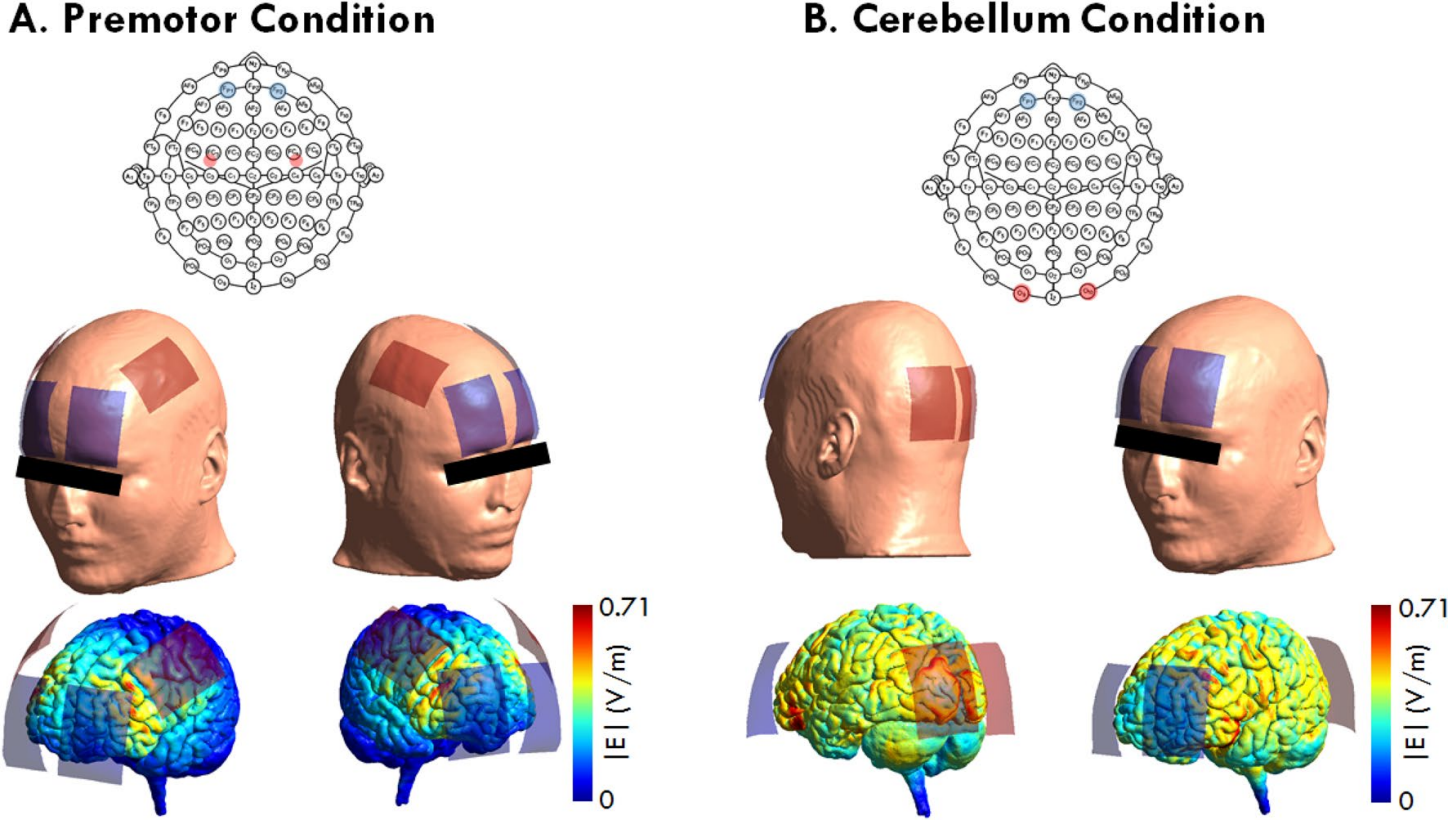
A tDCS-induced electrical fields (EFs) were estimated with a free and open source software package for simulation of non-invasive brain stimulation (SimNIBS v.3.2.3)^56^, using the default head model (ernie.msh). For the premotor stimulation sessions, anodal tDCS electrodes (size 35 cm^2^) were placed over the left and right premotor areas (2.5 cm anterior to the left and right M1 (C3 & C4) motor areas) and cathodal electrodes were placed over the Fp1 and Fp2 electrode positions. For the cerebellar stimulation sessions, the anodal electrodes (size 35 cm^2^) were placed over O9–O10 and the cathodal electrodes were placed over the Fp1 and Fp2 positions. Then the tDCS-induced EFs were estimated for 2 mA current intensity. Colors in the cortical grey matter are illustrating electric field magnitudes induced by tDCS (2 mA) estimated via SimNIBS open-source software with its default parameters and head model (ernie.msh).

TDCS was applied by a two channel electrical stimulator (NeuroStim 2, Medina Teb, Iran) using four saline-soaked (NaCl 0.9%) sponge electrodes. The electrode size was 35 cm^2^ (7 × 5 cm) and the stimulation duration was 20 min with a current intensity of 2 mA and 30 s ramping up and 30 s ramping down at the start, and end of stimulation. In both, pre-motor cortex and cerebellar conditions, the cathodal electrodes were placed over the FP1 and FP2 position, the anodal electrodes were placed bilaterally over the premotor cortex, or cerebellum according to the 10–20 International EEG System (O9-O10) (Fig. 2). Left and right premotor areas were defined as being 2.5 cm anterior to the left and right M1^49–51^. M1 was identified by C3 and C4 electrode positions of the International 10/20 EEG system^52^. For sham stimulation, the electrical current was ramped up for 30 s, followed by 15 s stimulation with 2 mA intensity, then ramped down for 30 s, and switched off without the subjects' knowledge to generate a sensation comparable to the real stimulation condition. In the sham condition, the target electrodes were randomly fixed over the premotor cortex for some participants and the cerebellum for others. Participants completed a survey regarding the tDCS side effects (e.g. itching, burning, and pain) on a 5-point Likert-scale at the end of each stimulation session^53^. Blinding efficacy was not explored. The experimenters who applied electrical stimulation were not blinded to the stimulation condition (i.e., sham vs active).

### Electromyography

Biometrics Ltd Surface EMG (sEMG) sensors and systems (Biometrics Ltd, UK) were used to record muscle activity during voluntary isometric contraction in the last 5 min of the tDCS intervention. EMG electrodes were placed on the main ventricle of the muscle and oriented over the following muscles (right and left sides): deltoid, biceps, triceps, trapezius, and pectoralis muscles according to the standard muscle map provided by the EMG software. In this study, we placed the sensor halfway the most distal motor endplate zone and the distal tendon of the muscle^54^. The skin under each electrode was shaved and cleaned with alcohol wipes before applying the EMG electrodes (Biometrics Ltd, for Windows, UK; electrode dimensions 38 × 20 mm, connected by two 4 mm snap connectors with 100 mm wires) over the muscles. The wireless transmitters and electrodes were fixed with elastic bands and EMG data were collected at 2,000 Hz.

### Computational modeling of tDCS-induced electrical fields

The tDCS-induced electrical fields (EFs) were estimated with a free and open source software package for simulation of non-invasive brain stimulation (SimNIBS v.3.2.3)^55^, using a default head model (ernie.msh). Briefly, this includes T1 image segmentation into the major head tissues, 3D volume reconstruction, placement of tDCS electrodes, assigning default tissue conductivities, and calculating the tDCS-induced EFs for 2 mA by means of the finite element method, under the quasi-static approximation^56^ (see Fig. 2).

### Procedure

The study had a randomized, single-blinded, sham-controlled cross-over design. Each elite gymnast participated in three tDCS conditions (i.e., premotor cortex stimulation, cerebellar stimulation, sham stimulation) in between-subject randomized order with one-week interval between sessions. Participants were asked to avoid high-intensity exercise or training for 48 h preceding the respective experimental session.

During each session, performance parameters were measured including broad jump, straddle lift to handstand, double legs circle, back hang scale, dips on parallel bars, active shoulder flexibility and sit and reach tests for 10 min before stimulation (1 min for the BJT test, 2 min for the SLHT test, 2 min for the DLCT test, 1 min for the BHST test, 1 min for the ASFT test, 1 min for the SRT test, and 2 min for DPBT test), then participants were seated on a comfortable chair and rested for 30 min until heart rate returned to resting rate. After electrode placement, tDCS was conducted for 20 min. During the last 5 min of tDCS intervention, 10 s EMG maximal isometric contraction signals were recorded for each muscle, after that the 5 s of the best MIVC were selected for each muscle. Then the performance parameters were obtained in the same way, including order, as for the baseline measures, for 10 min. In other words, performance parameters were measured offline (before and after intervention) and EMG parameters were measured online (during stimulation). All participants completed a standardized warm-up before these measures and a cool-down after the testing sessions. At the end of each session, participants were asked to complete a short survey about tDCS-related side effects (e.g. itching, burning, pain) on a 5-point Likert scale using a standard questionnaire^53^. Also the Borg rating of perceived exertion (RPE) scale related to physical activity intensity (e.g. no pain, mild, moderate, severe, very severe and worst pain possible) was conducted on a 10-point scale^57^.

### Statistical analysis

The SPSS statistical package, version 26.0 (IBM, SPSS, Inc., Chicago, IL) was used for data analysis. The normality of data distribution and homogeneity of variance were examined with Shapiro–Wilk and Levene tests. 2 × 3 repeated-measures ANOVAs were conducted with task-specific peak performance parameters (power speed (Broad Jump Test), strength/coordination (Straddle lift to handstand Test), endurance (Double Legs circle Test), static strength (Back Hang Scale Test), dynamic strength (Dips on Parallel Bars), flexibility (dynamic) (Active Shoulder Flexibility Test), flexibility (static) (Sit and Reach Test), and physical activity intensity (Rating of Perceived Exertion) as dependent variables, and time (pre-intervention, post-intervention) and stimulation condition (bilateral anodal tDCS over the pre-motor cortex; bilateral anodal tDCS over the cerebellum, and sham tDCS over the cerebellum or pre-motor cortex) as the within-subject factors. For EMG parameters, a within-subject repeated-measures ANOVA was conducted with stimulation condition as the within-subject factor. This measurement was conducted only during stimulation (last 5 min of stimulation) and thus no baseline was available. Before the ANOVA, Mauchly’s test was used to check the sphericity of the data. When this assumption was violated, a Greenhouse–Geisser correction was used. Conditional on significant results of the ANOVA, Fisher’s LSD post hoc tests (paired-sample, two-tailed) were performed for post hoc analysis.

## Results

### Data overview

**Figure 3:**
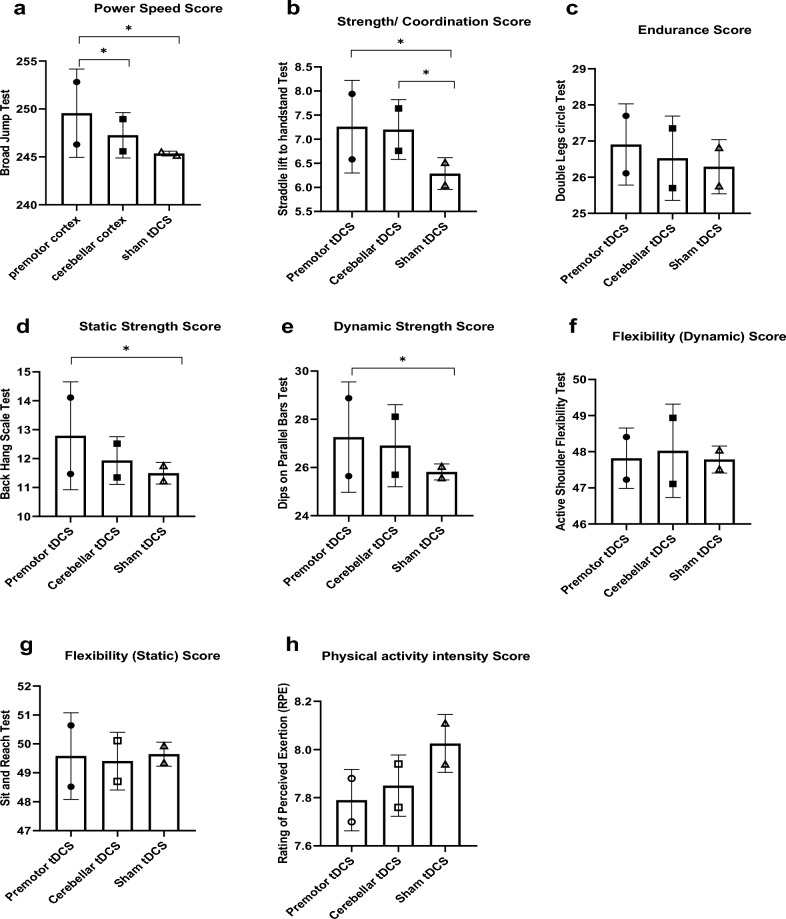
The impact of bilateral anodal cerebellar and premotor tDCS on peak performance parameters. Asterisks [*] represent a statistically significant difference between sham conditions and active protocols (premotor tDCS, cerebellar tDCS), and between active protocols before, and after stimulation. Filled symbols indicate significant pre to post differences within intervention conditions. *tDCS* transcranial direct current stimulation.

**Figure 4:**
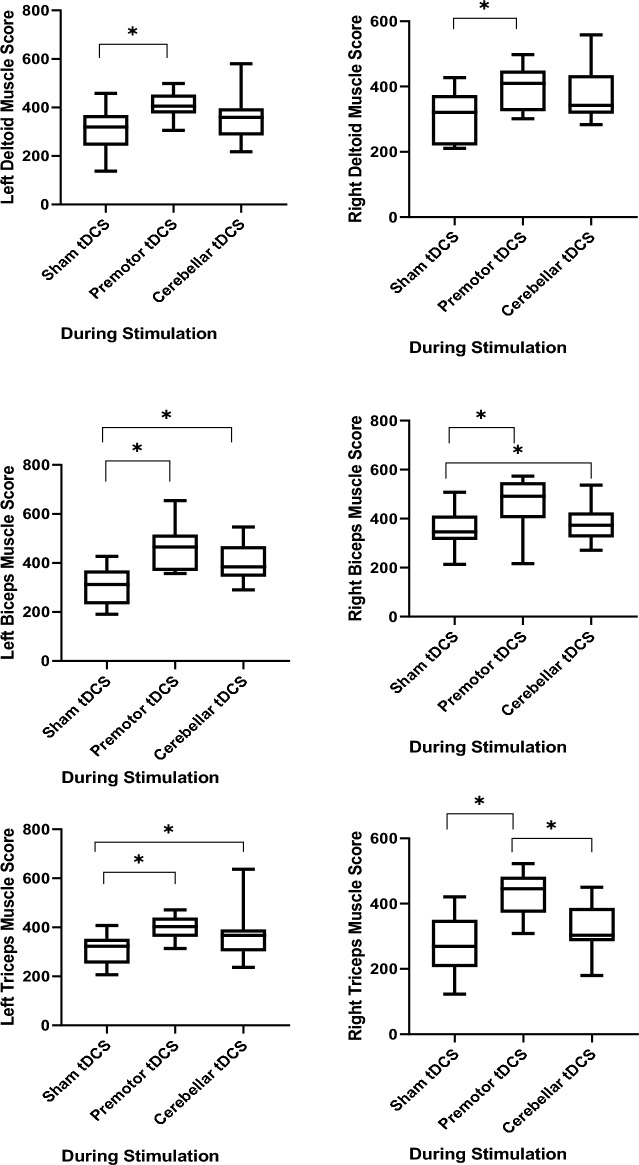

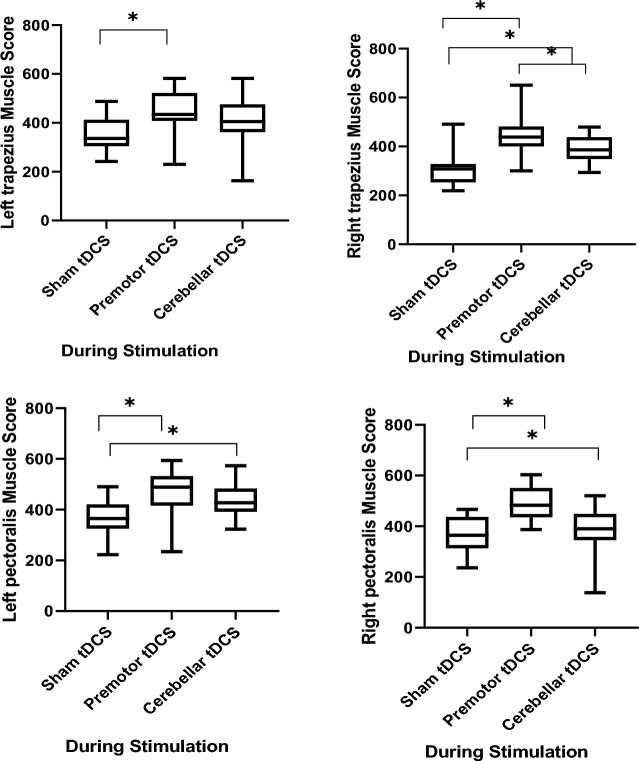
The impact of bilateral anodal cerebellar and premotor tDCS on online EMG parameters. Asterisks [*] represent a statistically significant difference between intervention conditions. *tDCS* transcranial direct current stimulation.

Participants tolerated the stimulation well and only mild side effects were reported (Table 2). For all side effects, real and sham stimulation conditions did not differ significantly (Burning sensation (F = 0.41, P = 0.91), Fatigue (F = 0.21, P = 0.81), Tingling (F = 2.13, P = 0.13), Pain, (F = 0.31, P = 0.72) and skin Redness (F = 2.13, P = 0.13). The data overview of peak performance before and after the intervention of active (premotor tDCS and cerebellar tDCS) and sham stimulation is presented in Tables 3, and 4 and Fig. 3. A data overview of the EMG parameters during active and sham stimulation is presented in Table 5 and Fig. 4.

**Table 2:** Means and SDs of the reported side effects during tDCS.

Variable	Outcome measures	Premotor tDCS	Cerebellar tDCS	Sham tDCS	F	df	p
		M (SD)	M (SD)	M (SD)			
tDCS side effects	Burning sensation	1.235 (0.437)	1.176 (0.392)	0.882 (0.696)	2.19	2	0.122
	Fatigue	0.117 (0.332)	0.117 (0.332)	0.058 (0.242)	0.211	2	0.811
	Tingling	0.117 (0.332)	0.000 (0.000)	0.000 (0.000)	2.13	2	0.130
	Pain	0.000 (0.000)	0.117 (0.000)	0.0352 (0.000)	0.314	2	0.725
	Redness	0.117 (0.332)	0.000 (0.000)	0.000 (0.000)	2.13	2	0.130

**Table 3:** Means and SD of peak performance before and after tDCS interventions.

Measure	Outcome variable	Time	Stimulation M (SD)		
			Premotor tDCS	Cerebellar tDCS	Sham tDCS
			M (SD)	M (SD)	M (SD)
Broad jump test	Power speed	Pre-intervention	246.29 (6.31)	245.58 (8.28)	245.17 (11.02)
		Post-intervention	252.82 (4.24)	248.94 (6.09)	245.52 (10.52)
Straddle lift to handstand test	Strength/coordination	Pre-intervention	6.58 (1.00)	6.76 (0.90)	6.52 (1.46)
		Post-intervention	7.94 (1.51)	7.64 (1.45)	6.05 (1.47)
Double legs circle Test	Endurance	Pre-intervention	26.11 (2.75)	25.70 (3.17)	25.76 (3.71)
		Post-intervention	27.70 (3.03)	27.35 (3.37)	26.82 (3.43)
Back hang scale test	Static strength	Pre-intervention	11.47 (2.26)	11.35 (2.08)	11.76 (2.63)
		Post-intervention	14.11 (2.78)	12.52 (2.40)	11.23 (2.22)
Dips on parallel bars	Dynamic strength	Pre-intervention	25.64 (2.59)	25.70 (3.07)	26.05 (4.40)
		Post-intervention	28.88 (2.86)	28.11 (3.44)	25.58 (3.26)
Active shoulder flexibility test	Flexibility (dynamic)	Pre-intervention	47.23 (3.78)	47.11 (3.95)	47.52 (5.51)
		Post-intervention	48.41 (4.92)	48.94 (3.81)	48.05 (5.00)
Sit and reach test	Flexibility (static)	Pre-intervention	48.52 (2.32)	48.70 (2.91)	49.35 (3.63)
		Post-intervention	50.64 (2.66)	50.11 (3.49)	49.94 (3.26)
Rating of perceived exertion (RPE)	Physical activity intensity	Pre-intervention	7.88 (0.69)	7.76 (0.66)	8.11 (0.69)
		Post-intervention	7.70 (0.46)	7.94 (0.65)	7.94 (0.74)

**Table 4:** Results of the repeated-measure ANOVAs for effects of stimulation (premotor tDCS, Cerebellar tDCS, and sham tDCS) and time (pre-intervention, post-intervention) on peak performance.

Measure	Outcome variable	Source	df	F	*p*-value	partial eta^2^
Broad jump test	Power speed	Time	1	33.64	< 0.001	0.71
		Stimulation	1.40,16	7.68	0.018	0.26
		Time × stimulation	1.48,32	5.44	< 0.001	0.62
Straddle lift to handstand test	Strength/coordination	Time	1	12.63	0.003	0.44
		Stimulation	2,16	5.90	0.007	0.27
		Time × stimulation	2,32	12.99	< 0.001	0.44
Double legs circle test	Endurance	Time	1	23.71	< 0.001	0.59
		Stimulation	2,16	0.852	0.436	0.05
		Time × stimulation	2,32	0.356	0.703	0.02
Back hang scale test	Static strength	Time	1	14.02	< 0.001	0.56
		Stimulation	2,16	3.17	0.021	0.24
		Time × stimulation	2,32	12.33	< 0.001	0.43
Dips on parallel bars	Dynamic strength	Time	1	41.32	< 0.001	0.81
		Stimulation	2,16	2.98	0.031	0.19
		Time × stimulation	2,32	16.38	< 0.001	0.58
Active shoulder flexibility test	Flexibility (dynamic)	Time	1	34.39	< 0.001	0.68
		Stimulation	2,16	0.298	0.744	0.01
		Time × stimulation	2,32	1.74	0.192	0.09
Sit and reach test	Flexibility (static)	Time	1	8.06	0.012	0.33
		Stimulation	2,16	0.090	0.914	0.06
		Time × stimulation	2,32	2.38	0.108	0.13
Rating of perceived exertion (RPE)	Physical activity intensity	Time	1	0.246	0.627	0.01
		Stimulation	2,16	0.980	0.386	0.05
		Time × stimulation	2,32	1.03	0.368	0.06

**Table 5:** Means and SDs of EMG parameters [maximum voluntary isometric contraction (MVIC)] during tDCS stimulation.

Measure	Outcome variable	Stimulation conditions			df	F	p-value	partial eta2
		Premotor tDCS	Cerebellar tDCS	Sham tDCS				
		M (SD)	M (SD)	M (SD)				
EMG	Left deltoid muscle	407.77 (53.73)	362.62 (89.01)	303.66 (84.03)	2	7.12	0.003	0.308
	Right deltoid muscle	395.00 (66.72)	379.97 (78.09)	306.42 (78.16)	2	7.32	0.002	0.314
	Left biceps muscle	457.75 (85.58)	403.99 (81.36)	303.69 (78.23)	2	18.44	< 0.001	0.536
	Right biceps muscle	471.63 (93.10)	375.06 (71.56)	355.51 (90.46)	2	8.97	< 0.001	0.359
	Left triceps muscle	396.81 (45.51)	364.69 (92.90)	306.79 (61.71)	2	6.20	0.005	0.279
	Right triceps muscle	430.35 (64.75)	327.83 (67.25)	279.15 (83.06)	2	20.03	< 0.001	0.556
	Left trapezius muscle	448.11 (86.40)	407.60 (96.87)	352.30 (67.08)	2	4.66	0.017	0.226
	Right trapezius muscle	451.92 (86.14)	387.00 (52.11)	304.72 (64.22)	2	16.28	< 0.001	0.504
	Left pectoralis muscle	462.16 (86.80)	432.01 (63.95)	364.74 (78.49)	2	8.23	< 0.001	0.340
	Right pectoralis muscle	489.51 (65.06)	384.16 (90.16)	367.58 (71.68)	2	8.18	< 0.001	0.454

### Efficacy of tDCS on peak performance

#### Power speed

The results of the respective two-way ANOVA conducted for the Broad Jump Test revealed significant main effects of stimulation (F1.40,16 = 7.68, p = 0.018, ηp2 = 0.26), time (F1,16 = 33.64, p < 0.001, ηp2 = 0.71) and a significant stimulation × time interaction (F1.48,32 = 5.44 p < 0.001, ηp2 = 0.62) on power speed scores. Post hoc comparisons revealed that bilateral anodal premotor stimulation significantly improved power speed as compared to bilateral anodal cerebellar stimlation (p = 0.034) and sham tDCS (p = 0.047) after intervention. No significant difference was observed between anodal cerebellar stimulation and sham tDCS (P = 0.477). Between-condition comparisons of power speed showed no significant difference between conditions in the pre-intervention measurement [PM vs sham (t = 0.844, p = 0.411), CB vs sham (t = 0.392, p = 0.700), PM vs CB (t = − 0.905, p = 0.379)]. Also comparisons between pre-to post intervention conditions showed no significant differences in the sham stimulation condition (t =  − 0.51, p = 0.611), but significant differences were obtained in the premotor (t =  − 8.96, p < 0.001) and cerebellar stimulation conditions (t =  − 4.73, p < 0.001).

#### Strength/coordination

For the straddle lift to handstand test, the ANOVA results showed a significant main effect of stimulation (F1,16 = 5.90, p = 0.007, ηp2 = 0.27), time (F1,16 = 12.63, p = 0.003, ηp2 = 0.44), and a significant stimulation × time interaction (F2,32 = 12.99, p < 0.001, ηp2 = 0.44) on strength/coordination scores. Post hoc comparisons revealed that both stimulation protocols, bilateral anodal premotor (p = 0.025) and bilateral anodal cerebellar tDCS (p = 0.017), significantly improved strength/coordination after intervention as compared to sham tDCS. No significant difference was observed between the two active stimulation protocols after intervention (p = 0.991). Between-condition comparisons of strength/coordination showed no significant difference between all conditons in the pre-intervention measurement (PM vs sham (t = 0.180, p = 0.859), CB vs sham (t = 0.846, p = 0.410), PM vs CB (t = − 0.677, p = 0.508)). Comparisons between pre-to post intervention conditions showed no significant differences in the sham stimulation (t = 2.05, p = 0.058), but significant performance enhancements in the premotor (t =  − 4.77, p < 0.001) and cerebellar stimulation conditions (t =  − 2.98, p = 0.009).

#### Endurance

For the double legs circle test, The results of the ANOVA showed no significant main effect of stimulation (F1,16 = 0.85, p = 0.436, ηp2 = 0.05), but a significant effect of time (F1,16 = 23.71, p < 0.001, ηp2 = 0.59), and no significant stimulation × time interaction (F2,32 = 0.35, p = 0.703, ηp2 = 0.02) on endurance scores). Between-condition comparisons of endurance showed no significant difference between all conditions in the pre-intervention measurement [PM vs sham (t = 0.752, p = 0.463), CB vs sham (t = -0.112, p = 0.912), PM vs CB (t = 0.891, p = 0.386)]. Comparisons between pre to post intervention conditions showed no significant differences in the sham stimulation (t = − 1.94, p = 0.070), but significant performance enhancements in the premotor (t =  − 2.85, p = 0.011) and cerebellar stimulation conditions (t =  − 2.98, p = 0.009).

#### Static strength

For the back hang scale test, the results of the ANOVA revealed a significant main effect of stimulation (F1,16 = 3.17, p = 0.021, ηp2 = 0.24), time (F1,16 = 14.02, p < 0.001, ηp2 = 0.56), and a significant interaction of stimulation × time (F2,32 = 12.33, p < 0.001, ηp2 = 0.43) on static strength scores. Post hoc comparisons revealed that bilateral anodal premotor tDCS significantly improved static strength as compared to sham tDCS (p = 0.042), but bilateral anodal cerebellar tDCS had no significant effect on static strength as compared to sham tDCS (p = 0.727). No significant difference was observed between the two active stimulation protocols (p = 0.539). Post hoc comparisons of static strength showed no significant difference between conditions in the pre-intervention measurements (PM vs sham (t = − 0.735, p = 0.473), CB vs sham (t = − 0.891, p = 0.386), PM vs CB (t = 0.293, p = 0.773)). Also comparisons between pre-to post intervention conditions showed no significant differences in the sham stimulation condition (t = 1.23, p = 0.236), but significant performance enhancements in the premotor (t =  − 7.09, p < 0.001), and cerebellar stimulation conditions (t =  − 3.51, p = 0.003).

#### Dynamic strength

With regard to the dips on parallel bars test, the results of the ANOVA showed a significant main effect of stimulation (F_1,16_ = 2.98, p = 0.031, ηp2 = 0.19), time (F_1,16_ = 41.32, p < 0.001, ηp2 = 0.81) and a significant interaction of stimulation × time (F_2,32_ = 16.38, p < 0.001, ηp2 = 0.58) on dynamic strength scores. Post hoc comparisons showed that bilateral anodal premotor tDCS significantly improved dynamic strength as compared to sham tDCS (p = 0.048), but bilateral anodal cerebellar tDCS had no significant effect on dynamic strength as compared to sham tDCS (p = 0.158). No significant difference was observed between the two active stimulation protocols (p = 0.981). Post hoc comparisons of dynamic strength showed no significant difference between conditions in the pre-intervention measurement [PM vs sham (t = − 0.630, p = 0.537), CB vs sham (t = − 0.607, p = 0.552), PM vs CB (t = − 0.117, p = 0.908)]. Comparisons between pre-to post intervention conditions showed no significant differences in the sham stimulation (t = 0.91, p = 0.375), but significant performance enhancements in the premotor (t =  − 10.25, p < 0.001) and cerebellar stimulation conditions (t =  − 7.50, p = 0.001).

#### Dynamic flexibility

For dynamic flexibility (Active Shoulder Flexibility Test), the results of the ANOVA showed no significant main effect of stimulation (F_2,16_ = 0.298, p = 0.724, ηp2 = 0.01) but a significant main effect of time (F_1,16_ = 34.39, p < 0.001, ηp2 = 0.68) and no significant stimulation × time interaction (F2,32 = 1.74, p = 0.192, ηp2 = 0.09). Between-condition comparisons of dynamic flexibility showed no significant difference between all conditions in the pre-intervention measurement [PM vs sham (t = − 0.411, p = 0.687), CB vs sham (t = − 0.620, p = 0.544), PM vs CB (t = 0.226, p = 0.824)]. Comparisons between pre-to post intervention conditions showed no significant differences in the sham stimulation (t = − 0.94, p = 0.361), but significant performance enhancements in the premotor (t =  − 3.54, p = 0.003) and cerebellar stimulation conditions (t =  − 3.00, p = 0.008).

#### Static flexibility

For static flexibility (Sit and Reach Test), the results of the ANOVA showed no significant main effect of stimulation (F_2,16_ = 0.090, p = 0.914, ηp2 = 0.06), but a significant main effect of time (F_1,16_ = 8.06, p = 0.012, ηp2 = 0.33) and no significant interaction of stimulation × time (F_2,32_ = 2.38, p = 0.108, ηp2 = 0.13). Between-condition comparisons of static flexibility showed no significant difference between all conditions in the pre-intervention measurement [PM vs sham (t = − 1.42, p = 0.173), CB vs sham (t = − 1.10, p = 0.287), PM vs CB (t = − 0.387, p = 0.704)]. Comparisons between pre-to post intervention conditions showed no significant differences in the sham stimulation (t = − 0.82, p = 0.421), but significant performance enhancements in the premotor (t =  − 3.96, p < 0.001) and cerebellar stimulation conditions (t =  − 2.23, p = 0.040).

#### Physical activity intensity

Finally, For physical activity intensity (Rating of Perceived Exertion), the results of the ANOVA showed no significant main effects of stimulation (F_2,16_ = 0.980, p = 0.386, ηp2 = 0.05), time (F_1,16_ = 0.246, p = 0.627, ηp2 = 0.01), and the stimulation × time interaction (F_2,32_ = 1.03, p = 0.368, ηp2 = 0.06). Between-condition comparisons of physical activity showed no significant difference between all conditons in the pre-intervention measurement [PM vs sham (t = − 1.28, p = 0.216), CB vs sham (t = − 1.37, p = 0.188), PM vs CB (t = 0.416, p = 0.683)].

### Effects of tDCS on EMG parameters

The results of the repeated-measures ANOVAs showed a significant main effect of stimulation on the EMG parameters of all muscles, including the left deltoid muscle (F_2,16_ = 7.12, p = 0.003, ηp2 = 0.30), right deltoid muscle (F_2,16_ = 7.32, p = 0.002, ηp2 = 0.31), left biceps muscle (F_2,16_ = 18.44, p < 0.001, ηp2 = 0.53), right biceps muscle (F_2,16_ = 8.97, p < 0.001, ηp2 = 0.35), left triceps muscle (F_2,16_ = 6.20, p = 0.005, ηp2 = 0.27), right triceps muscle (F_2,16_ = 20.03, p < 0.001, ηp2 = 0.55), left trapezius muscle (F_2,16_ = 4.66, p = 0.017, ηp2 = 0.22), right trapezius muscle (F_2,16_ = 16.28, p < 0.001, ηp2 = 0.50), left pectoralis muscle (F_2,16_ = 8.23, p < 0.001, ηp2 = 0.34) and right pectoralis muscle (F_2,16_ = 13.31, p < 0.001, ηp2 = 0.45).

Post hoc comparisons revealed that bilateral anodal premotor tDCS significantly increased MVIC of the left deltoid muscle as compared to sham tDCS (p = 0.004). For the right deltoid muscle, both premotor (p = 0.009) and cerebellar (p = 0.020) tDCS significantly increased MVIC compared to sham tDCS. No significant difference was observed between the two active stimulation protocols (p > 0.998).

For the left biceps muscle, again both, premotor cortex (p < 0.001) and cerebellar cortex (p < 0.001) stimulation significantly increased MVIC compared to sham tDCS. No significant difference was found between the active stimulation protocols (p = 0.239). For the right biceps muscle, bilateral anodal stimulation of the premotor cortex significantly increased MVIC compared to cerebellar stimulation (p = 0.011) and sham tDCS (p = 0.007). For the left triceps muscle, stimulation of the premotor cortex increased MVIC as compared to sham tDCS (p = 0.003). For the right triceps muscle, stimulation of the premotor cortex significantly increased MVIC compared to cerebellar cortex stimulation (p < 0.001) and sham tDCS (p < 0.001). Premotor cortex stimulation significantly increased MVIC of the left trapezius muscle compared to sham tDCS (p = 0.016), and for the right trapezius muscle, both premotor (p < 0.001), and cerebellar (p = 0.007) stimulation significantly increased MVIC compared to sham tDCS. Finally, for the left pectoralis muscle, both, premotor (p = 0.011) and cerebellar (p = 0.031) stimulation significantly increased the MVIC index compared to sham tDCS, and for the right pectoralis muscle, premotor tDCS significantly increased MVIC compared to cerebellar cortex stimulation (p < 0.001) and sham tDCS (p = 0.006).

## Discussion

In this randomized, cross-over, sham-controlled study, we investigated the impact of bilateral anodal tDCS (2 mA, 20 min) over the premotor and cerebellar cortices on peak performance and physiological parameters in a sample of professional gymnastic athletes. The results showed that in general premotor and/or cerebellar tDCS improved performance parameters (e.g., power speed, static and dynamic strength) as compared to sham, and EMG parameters showed an enhancement of MVIC in right and left upper limb muscles. These results support our hypothesis about the important role of premotor and cerebellar cortices in improving performance parameters in professional gymnastic athletes.

With respect to task performance, bilateral anodal premotor stimulation furthermore significantly improved power speed, strength/coordination, as well as static and dynamic strength, and bilateral anodal cerebellar stimulation improved strength/coordination as compared to sham tDCS. Moreover, bilateral anodal premotor stimulation significantly improved power speed in the broad jump test, as compared to bilateral anodal cerebellar stimulation. Comparisons between pre-to post-intervention conditions in all tasks showed no significant differences in the sham stimulation condition, but significant performance enhancements in the premotor and cerebellar stimulation conditions in all tasks except Rating of Perceived Exertion (for physical activity intensity). However, neither type of tDCS had a significant effect on endurance scores, static or dynamic flexibility and physical activity intensity factors. In general, consistent with these findings, in sports, bilateral M1 tDCS-induced improvements in athletic performance with regard to cycling endurance performance^43^ have been reported, as well as improvement of unilateral single-joint movement^58^ and jumping performance^29^ by anodal motor cortex tDCS. On the other hand, in contrast to the findings of the present study, anodal bi-hemispheric stimulation over M1 worsened the performance of taekwondo athletes in the Frequency Speed of Kick Test (FSKT) and RPE, and this effect remained even for 1 h after stimulation^59^. With respect to studies exploring the role of cerebellar stimulation in sports-specific performance, we found only one available study. Kamali et al. showed that shooting accuracy of pistol shooters increased with cerebellar anodal stimulation^60^. The authors attributed this effect to improved postural adaptation that allowed shooters to reduce physiological tremor. Our findings are furthermore supported by previous studies that suggest that tDCS over the cerebellum and M1 enhances motor learning^61^. Also, imaging data during cerebellar tDCS showed an increase in learning-specific activity in areas related to the motor learning network^61^.

For a mechanistic explanation of these effects, it has been shown that anodal tDCS over M1 enhances functional connectivity of the cortico-cortical, and cortico-subcortical motor networks^28^. It is likely that this network activity-enhancing effect takes place also for premotor stimulation, and results in performance improvement. Motor network tDCS, which is related to our bilateral premotor stimulation approach, has been shown to have more profound effects than single target motor cortex tDCS on cortical excitability^62^. It seems that bilateral stimulation has better performance effects than unilateral stimulation that can be study in future studies. With respect to the involvement of the cerebellum, resting-state connectivity studies in healthy subjects have shown enhanced connectivity in fronto-parietal-cerebellar networks^63,64^ as well as motor cortex-cerebellar networks^65^ following motor adaptation. These results suggest that also cerebellar activation via tDCS might exert its performance-enhancing effects via motor network activation. At the cellular level, LTP-like plasticity induced by anodal tDCS is most likely the driving force of the effects on performance, since we performed tDCS before task performance, and thus acute polarization effects will not be present during task performance. This plasticity can indeed enhance motor network activity^28^, and also specifically premotor-motor connectivity^66^, and similar effects might be valid also for cerebellar stimulation. However, inhibitory interactions between the cerebellum and M1, cerebellum-brain inhibition (CBI)^67^, might have limited cerebellar effects on performance. One of the important and unresolved questions is the neurophysiological mechanism underlying the enhanced effects of bilateral stimulation. A probable explanation is that in our study we applied bilateral tasks, which require activation of bilateral motor networks for maximum efficacy, which might be accomplished by bilateral excitability-enhancing stimulation.

In accordance with our behavioral results, for the EMG parameters, bilateral premotor stimulation improved the MVIC index in all upper limb muscles (bilateral deltoid, biceps, triceps, trapezius, and pectoralis muscles), as compared to sham stimulation, while bilateral cerebellar anodal stimulation improved MVIC as compared to sham only in some of these muscles (right deltoid, left biceps, right trapezius and left pectoralis muscles). Furthermore, for some of the tested muscles, real stimulation over the premotor cortex had larger effects as compared to cerebellar stimulation, suggesting a superiority of premotor over cerebellar tDCS (Fig. 3). These basic effects are in accordance with the performance enhancements discussed above, including the superior effects of premotor stimulation, and can be mechanistically explained by anodal tDCS-induced excitability enhancement, in this case via neuroplastic, but also acute effects of stimulation, as MVIC measures were obtained during stimulation. A recent study^68^ applied anodal tDCS over M1 for 20 min at 2 mA intensity and found increased spontaneous EMG activity in the biceps brachii muscle after active tDCS, as compared to baseline EMG, supposedly caused by M1 activity enhancement generated by tDCS. Similarly, Hazime et al. showed that anodal tDCS over M1 contralateral to the dominant limb with an intensity of 2 mA for 20 min increased MIVC of the internal and external rotators of the shoulder in handball players^69^. Therefore, an increased number of recruited muscle fibers^70^ or increased firing rates of motor units^70^ due to synchronization of motor units based on enhanced excitability of the M1 and/or the corticospinal tract^71^ during MIVC is a potential mechanism of tDCS effects on muscle activity. The minor effect of cerebellar stimulation might be again caused by motor cortex inhibition via cerebellar stimulation, as outlined above, In contrast to these findings, Flood et al. describe no increase of maximal force production by anodal HD-tDCS placement in a 4 × 1 ring configuration with the centre electrode positioned on the scalp over the hand motor cortex contralateral to the non-dominant side (C3/C4) and return electrodes positioned in a ring around the centre anode at a radius of approximately 5 cmcorresponding to Cz, F3/F4, T7/T8 and P3/P4 in healthy subjects^72^. A possible reason for the missing effects in that study might be that presumably more focalized stimulation in combination with a not physiologically determined hotspot would have resulted in a larger probability to miss the motor cortex representation of the target muscles, as compared to a conventional approach with larger electrodes.

Target region and stimulation intensity are two important factors which determine stimulation efficacy^73^. To our knowledge, this is the first study that investigated the impact of bilateral anodal stimulation over the premotor and cerebellar cortices on physiological (muscle strength) and performance parameters of gymnastic athletes. Most previous studies investigated unilateral stimulation over the left M1 (C3)^69,74^, the right M1 (C4)^58,69,74^, the left temporal cortex^75,76^, left and right dorsal premotor cortex^77^, and cerebellum^61^. The main advantage of the present study, as compared to most previous ones, is the bilateral stimulation approach.

Comparing tDCS over the premotor cortex and cerebellum, previous studies have demonstrated a role of both areas in motor learning^78,79^. However, in the present study we observed in general more prominent effects of premotor stimulation. Some reasons for this pattern of results are the following. The cerebellum is most prominently involved in ballistic and coordination tasks^80^ whereas the premotor cortex has a more general relevance for posture and movement planning ^81^. This might explain the effects of premotor stimulation on a broader range of tasks in the present study. Second, effect differences, with partially larger effects of premotor stimulation can be explained based on cerebellar connectivity. Anodal tDCS over the cerebellum has been shown to increase cerebellar brain inhibition, which means that it reduces primary motor cortex excitability^82^, and thus might limit the effects on motor performance, as compared to premotor stimulation. Moreover, it has been postulated that while the cerebellum is primarily important for the early stage of memory formation via error-based learning, the motor cortex is more relevant for the retention of motor memories during the late stage of learning, and consolidation^83^, the latter being more relevant for the tasks conducted in the present study.

Our study has several implications. First of all, we showed that the use of bilateral anodal stimulation with 2 mA intensity was well tolerated and elicited no serious side effects. Second, we showed that bilateral anodal tDCS over the premotor cortex and cerebellar tDCS is effective for improving peak performance parameters in gymnastic athletes.

## Limitations and future directions

This work has some limitations, which should be taken into account. This is a single-session proof-of-principle study, which did not aim to optimize stimulation protocols to achieve maximal effects. Adaptation of the protocol, including stimulation area, intensity, duration, number of sessions, and adaptation of the intervention protocol to task characteristics might enhance efficacy further. In previous studies, multi-session compared to single-session interventions have been shown to be more efficient^84^. Another limitation of this study is its small sample size. Future studies should also investigate the effect of tDCS on physiological parameters to a larger degree, including functional measures more closely related to competitive sports. Given the suggested suggestibility of athletes for expectency effects, future studies should should have preferentially a double-blinded design. Another limitation of the present study is the large size of the electrodes, which most likely includes the stimulation area not only the premotor area, but probably the primary motor cortex and the supplementary area. Therefore, it is suggested for future researches to use smaller size electrodes or HD-tDCS method to localize the stimulation.

## Conclusion

We investigated the effects of bilateral tDCS over the premotor cortex and cerebellum on physiological functions (MVIC for bilateral deltoid, biceps, triceps, trapezius, and pectoralis muscles) and peak task performance of professional gymnastics athletes. The current findings show effects of stimulation over both areas for task performance, and MIVC, but superior stimulation effects over the premotor region compared to cerebellum on improving peak performance level. Compared to sham stimulation, stimulation of the cerebellum improved performance just in the straddle lift to handstand test, which measures strength/coordination. Furthermore, premotor tDCS impacted a larger number muscles with respect to MIVC than cerebellar stimulation, and premotor effects were larger than cerebellar effects in some muscles. Thus the results of this proof-of-concept study hold promise. More knowledge about respective mechanisms of action, as well as systematic optimizing studies to enhance efficacy, and applicability of this intervention are required.

## Data Availability

The datasets used and/or analysed during the current study available from the corresponding author on reasonable request.
